# DEPTOR is linked to a TORC1-p21 survival proliferation pathway in multiple myeloma cells

**DOI:** 10.18632/genesandcancer.44

**Published:** 2014-11

**Authors:** Yonghui Yang, Carolyne Bardeleben, Patrick Frost, Bao Hoang, Yijiang Shi, Richard Finn, Joseph Gera, Alan Lichtenstein

**Affiliations:** ^1^ Department of Medicine, Hematology-Oncology, Greater Los Angeles VA Healthcare Center, Los Angeles, CA, USA; ^2^ UCLA School of Medicine, Greater Los Angeles VA Healthcare Center, Los Angeles, CA, USA; ^3^ Jonsson Comprehensive Cancer Center, Greater Los Angeles VA Healthcare Center, Los Angeles, CA, USA

**Keywords:** Multiple Myeloma, DEPTOR, mTORC1, ER stress, AKT, p21

## Abstract

We investigated the mechanism by which gene silencing of the mTOR inhibitor, DEPTOR, induces cytoreductive effects on multiple myeloma (MM) cells. DEPTOR knockdown resulted in anti-MM effects in several MM cell lines. Using an inducible shRNA to silence DEPTOR, 8226 MM cells underwent TORC1 activation, downregulation of AKT/SGK activity, apoptosis, cell cycle arrest and senescence. These latter cytotoxic effects were prevented by TORC1 paralysis (Raptor knockdown) but not by over-expression of AKT activity. In addition, DEPTOR knockdown-induced MM death was not associated with activation of the unfolded protein response, suggesting that enhanced ER stress did not play a role. In contrast, DEPTOR knockdown in 8226 cells induced p21 expression, independent of p53, and p21 knockdown prevented all of the cytotoxic effects following DEPTOR silencing. DEPTOR silencing resulted in p21 upregulation in additional MM cell lines. Furthermore, DEPTOR silencing in a murine xenograft model resulted in anti-MM effects associated with p21 upregulation. DEPTOR knockdown also resulted in a decreased expression of p21-targeting miRNAs and transfection of miRNA mimics prevented p21 upregulation and apoptosis following DEPTOR silencing. Use of a shRNA-resistant DEPTOR construct ruled out off-target effects of the shRNA. These results indicate that DEPTOR regulates growth and survival of MM cells via a TORC1/p21 pathway and suggest an involvement of p21-targeted miRNAs.

## INTRODUCTION

DEPTOR is a recently described 48 kDa protein that binds to mTOR and inhibits this kinase within TORC1 and TORC2 complexes [1]. Over-expression of DEPTOR specifically occurs in the multiple myeloma (MM) tumor model. The highest levels of expression are found in the genetic category of MM that is non-hyperdiploid and, more specifically, in those myelomas that contain translocations between the IgH and MAF or cyclin D1 genes with dysregulated MAF or cyclin D-1 levels [1,2]. DEPTOR is a transcriptional target of MAF [1] which probably explains its heightened expression in MAF-translocated MM cases. The mechanism of DEPTOR upregulation in cyclin D-1-translocated MM is unknown but may relate to frequent copy number gains and increased expression of genes at chromosome 8q24 [3], a region which contains DEPTOR. Most interestingly, DEPTOR knock-down (KD) in over-expressing MM cell lines induces growth arrest and apoptosis, indicating DEPTOR could serve as a therapeutic target in selected patients [1].

The mechanism by which DEPTOR knock-down induces MM death is unknown but one possibility is via a feedback inhibition of the PI3K/AKT/SGK cascade. Activation of mTOR within TORC1 results in feedback inhibition of PI3K by several different mechanisms [4-7]. This occurs in many different tumor types as well as in MM [7]. Thus, acute activation of TORC1 by DEPTOR KD may kill MM cells by downregulation of the PI3K/AKT survival pathway. A second possibility, however, could be that, by activating TORC1 and acutely stimulating cap-dependent translation, DEPTOR KD rapidly enhances ER stress to which MM cells are particularly susceptible [8] because of their role in Ig synthesis. It is also possible that a combination of enhanced ER stress and inhibited PI3K/AKT work together to induce MM cell death. A final possibility, of course, is that DEPTOR KD induces anti-MM effects by yet identified mechanisms that could be mTOR-dependent or –independent.

In this mechanistic study, we investigated anti-MM effects following DEPTOR silencing. Neither AKT inhibition nor ER stress induction could explain the anti-MM effects. In contrast, a novel TORC1-miRNA-p21 pathway was implicated.

## RESULTS

### DEPTOR silencing results in MM cell apoptosis, cell cycle arrest and senescence

**Figure 1:**
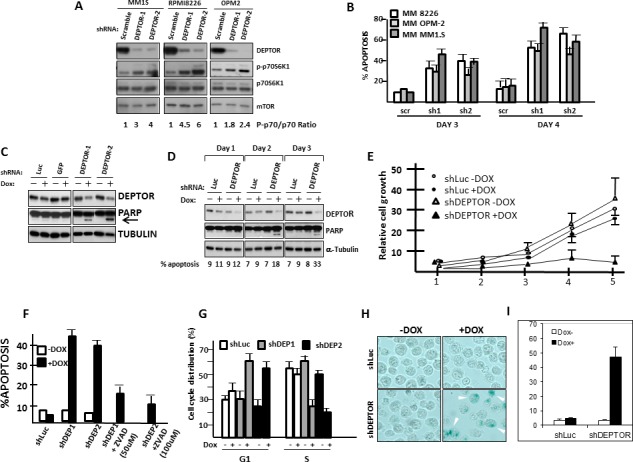
DEPTOR knockdown induces Apoptosis, G1/S cell cycle arrest & senescence in MM cells A). Cell lines acutely infected with control (scramble) or two separate DEPTOR shRNAs followed by Western blot. Phospho-P70/total P70 ratio shown under the gel was determined by densitometry and is mean of 3 separate experiments; B) % apoptosis 3 or 4 days after lentivirus infection with control (scr) or shRNA to DEPTOR (sh1, sh2). C) 8226 transfected with dox-inducible control shRNA (Luc or GFP) or shRNA targeting 2 sequences of DEPTOR (DEPTOR-1/DEPTOR-2); Dox added to induce shRNAs and immunoblot 72 hrs later for DEPTOR expression or PARP cleavage (arrow); D) Immunoblot assay performed in 8226 cells at 1, 2 or 3 days after adding dox; E) MTT assay over time after adding dox to induce shRNA; F) Apoptosis assay at 72 hrs (flow analysis of activated caspase 3) in control (shLuc) or DEPTOR knockdown cells (shDEP) +/− dox. ZVAD added at 50 or 100 uM. G) G1 or S distribution in transfected 8226+/− dox. H) Senescence assay (SA-beta gal staining (arrows)) in DEPTOR knockdown cells after adding dox; I) Quantification of senescence. All data in figs 1B, 1E, 1F, 1G and 1I represent means+/−SD, n=3).

The highest levels of DEPTOR mRNA are found in specific genetic categories of multiple myeloma [1]. In contrast, in most other malignancies, DEPTOR expression levels are lower than corresponding normal tissues [1]. This suggests that DEPTOR plays a singular role in myelomagenesis and the finding that DEPTOR knockdown (KD) in the 8226 MM cell line is cytotoxic [1] supports this notion. To confirm our different MM cell lines are affected similarly by DEPTOR KD, we silenced DEPTOR with shRNA targeting two distinct sequences in the DEPTOR-over-expressing MM1.S, OPM-2 and 8226 MM lines. Since DEPTOR is an mTOR inhibitor, its silencing is expected to increase TORC1 activity and this was monitored by assessing phosphorylation of p70S6K. TORC1 activity was indeed increased in all three DEPTOR-silenced MM lines (phospho-p70/total p70 ratio, below gel in Fig 1A). All three MM lines underwent significant apoptosis following DEPTOR KD (Fig 1B).

To further study the mechanism by which DEPTOR KD is cytotoxic to MM cells, we used doxycycline-inducible shRNAs. The inducible shRNAs made it more likely that molecular alterations seen early after doxycycline (dox) induction were proximal effects of DEPTOR KD rather than secondary effects of MM cell apoptosis/growth arrest. As shown in fig 1C, both shRNA in 8226 MM cells resulted in significant DEPTOR silencing upon addition of dox while dox added to empty vector control cells (“luc” and “GFP” in fig 1C) had no effect. At this time point (72 hrs after induction) we could also detect PARP cleavage (arrow in fig 1C), implicating an apoptotic response only in 8226 cells transfected with DEPTOR shRNA and exposed to dox. A time course experiment (fig 1D) demonstrated that inhibited DEPTOR protein expression was already detected by 24 hrs after dox addition but significant PARP cleavage required 48 hrs. An MTT assay (fig 1E) demonstrated that shRNA targeting of DEPTOR significantly inhibits 8226 growth *in vitro*. Fig 1E depicts data from shRNA targeting sequence 1(DEPTOR-1). Targeting to the DEPTOR-2 sequence likewise resulted in depressed 8226 growth (suppl fig 1).

Flow cytometric assessment of apoptosis (activated caspase 3 activity, fig 1F) after 72 hrs of dox exposure clearly demonstrated that shRNA targeting both sequences of DEPTOR resulted in significant apoptosis. Similar to the development of PARP cleavage, the kinetics of apoptosis induction assessed by caspase 3 activation was also first detected at 48 hrs (% apoptosis shown below gel of fig 1D). As expected, apoptosis was inhibited by the ZVAD caspase inhibitor (fig 1F). In addition to apoptotic death, DEPTOR knockdown also induced cell cycle arrest (fig 1G), shown by an increase in G1 and decrease in S phase distribution. Finally, 8226 cells also underwent senescence following DEPTOR KD as shown microscopically in fig 1H and quantitatively in fig 1I. Collectively, these data confirm the adverse effects of DEPTOR KD in MM cells with the induction of apoptosis, G1/S arrest and senescence.

### Molecular effects of DEPTOR knockdown in the inducible 8226 MM cell line

**Figure 2:**
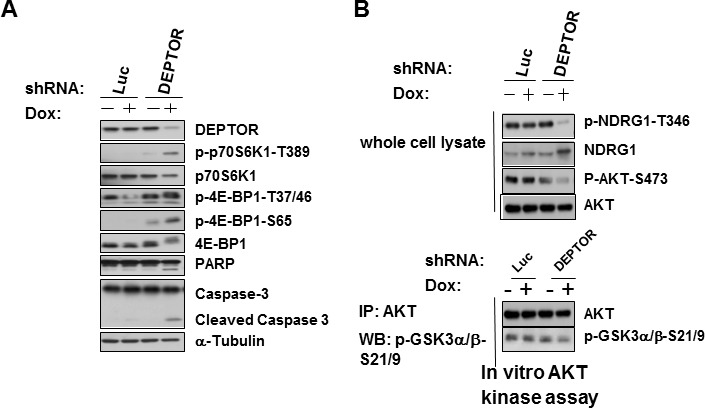
Molecular effects of DEPTOR knockdown in 8226 cells A) Control (Luc) or DEPTOR knocked down cells +/− dox, followed by Western blot; B)Extract from same cells as in ‘A’ immunoblotted for NDRG-1 or phosphorylated AKT. AKT also immunoprecipitated from extracts and tested for kinase activity against GSK (bottom).

Upon dox-induced DEPTOR KD in 8226 cells, TORC1 activity was stimulated as expected. This was shown as an upregulation of p70S6K phosphorylation on T389 in dox-treated shRNA cells (Fig 2A). The other TORC1 substrate, 4E-BP1, also demonstrated enhanced phosphorylation on T37/46 and S65. Total levels of p70S6K and 4E-BP1 were not affected although the electrophoretic migration of 4E-BP1 was altered (Fig 2A), probably representing enhanced phosphorylation. PARP cleavage and proteolytic activation of caspase 3 is again shown in fig 2A upon DEPTOR silencing. As described above [4-7], TORC1 stimulation results in feedback inhibition of the PI3K/AKT/SGK pathway. Thus, it was not surprising that DEPTOR knockdown with ensuing TORC1 stimulation was also associated with inhibition of AKT and SGK activity. To assay SGK activity we performed immunoblot for phosphorylation of NDRG-1, an SGK substrate (Fig 2B). As shown, DEPTOR knock-down resulted in a significant decrease in phosphorylation of NDRG-1 on T346. Immunoblot assay also demonstrated inhibited phosphorylation of AKT on S473 following DEPTOR knockdown (fig 2B) as well as decreased AKT *in vitro* kinase activity against GSK (lower panel of fig 2B). Thus, we could reproduce the negative feedback inhibition resulting from DEPTOR silencing previously described by Peterson et al [1].

### Effects of RAPTOR silencing on MM cell death induced by DEPTOR knockdown

**Figure 3:**
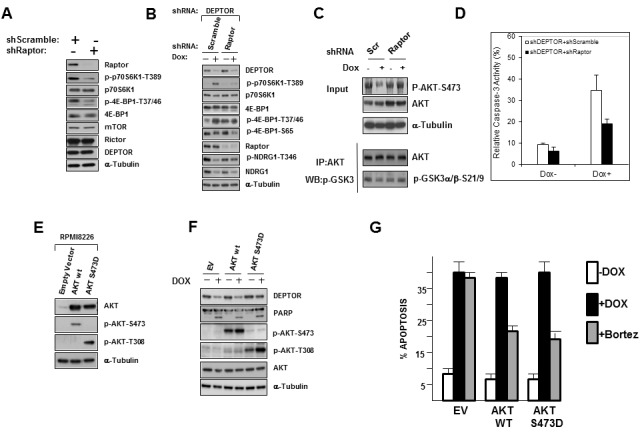
Apoptosis induced by DEPTOR knockdown is TORC1-dependent but independent of AKT inhibition A) Inducible shRNA cell line infected with shcontrol (scramble) or shRNA targeting RAPTOR followed by Western blot; B)Same cells as in ‘A’ treated +/− dox to induce DEPTOR silencing, followed by Western Blot; C)Same cells as in ‘A’ or ‘B’, treated +/− dox, followed by Western blot for phospho-AKT or in AKT vitro kinase activity against GSK (bottom); D) DEPTOR knockdown-induced apoptosis is prevented by RAPTOR knockdown (mean+/−SD, n=4, RAPTOR knockdown significantly (p<0.05) inhibited dox-induced apoptosis); E) Effects of ectopic expression of wild type (WT) AKT or phosphomimetic AKT (AKT S473D); F) Same cells as in ‘E’ treated +/− dox to silence DEPTOR, followed by Western blot. G) DEPTOR knockdown induction of apoptosis is unaffected by ectopic over-expression of AKT (mean+/−SD, n=3) although bortezomib-induced apoptosis is significantly inhibited (p<0.05).

To test if the adverse effects of DEPTOR KD in MM cells were mediated via TORC1 stimulation, we infected our dox-inducible shRNA-transfected 8226 cells with shRNA to silence RAPTOR or, as a control, directed towards a scrambled sequence. RAPTOR knockdown was efficient as shown in fig 3A. In the absence of dox (ie., no DEPTOR silencing), RAPTOR knockdown resulted in downregulation of TORC1-induced phosphorylation of p70S6K and 4E-BP1 but had no effect on RICTOR, mTOR or DEPTOR expression (Fig 3A). When dox was added to these cells to additionally silence DEPTOR, RAPTOR knockdown prevented the expected upregulation of TORC1 activity (fig 3B). As shown, dox-induced DEPTOR knockdown in scramble-transfected cells resulted in the anticipated stimulation of p70S6K and 4E-BP1 phosphorylation. However, concurrent RAPTOR knockdown prevented these increases. Immunoblot for phospho-NDRG-1 in these cells also demonstrated that RAPTOR knockdown prevented the feedback inhibition of the PI3K/SGK/AKT pathway resulting from DEPTOR silencing. Feedback inhibition of AKT activity was also prevented by RAPTOR KD as shown by immunoblot assay (fig 3C) for phosphorylated AKT (on S473) as well as *in vitro* AKT kinase activity. RAPTOR silencing also significantly prevented MM cell apoptosis resulting from DEPTOR KD (fig 3D), indicating that effects downstream of TORC1 stimulation mediate the negative effects on MM cells.

### Effects of constitutive AKT activation

To directly test if the feedback inhibition of AKT resulting from DEPTOR knockdown was implicated in MM cell death, we ectopically expressed wild type (WT) AKT or a phosphomimetic version (S473D) into our DEPTOR shRNA MM cells. As shown in fig 3E, the WT AKT-transfected cells demonstrated enhanced levels of AKT phosphorylated on S473 compared to EV-transfected cells. The phosphomimetic AKT-transfected cells demonstrated high levels of AKT phosphorylation on T308 while, as expected, the S473 phospho-AKT antibody could not detect the mutated residue at 473. When these cells were treated with dox to induce DEPTOR knockdown, phosphorylation of the transfected AKT versions is maintained and not decreased by DEPTOR knockdown (fig 3F). However, this over-expression of AKT is not capable of preventing apoptosis induced by DEPTOR knockdown (fig 3G). The anti-apoptotic potential of the transfected AKT constructs (WT and phosphomimetic versions) is shown by their ability to inhibit MM cell apoptosis induced by bortezomib (fig 3G). It is, thus, clear that the downregulation of AKT activity, induced by DEPTOR knockdown and its resulting negative feedback loop, does not contribute to the apoptotic response.

### DEPTOR knockdown does not induce ER stress

**Figure 4:**
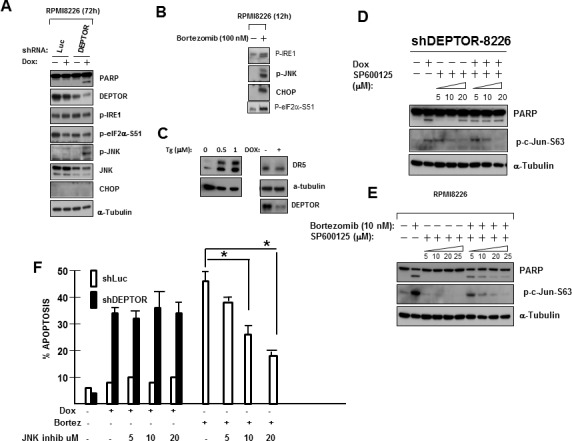
DEPTOR knockdown-induced Apoptosis is not due to heightened ER stress A) shRNA-transfected 8226 cells treated +/− dox followed by immunoblot; B) DEPTOR shRNA-transfected 8226 cells treated +/− bortezomib, followed by Western blot; C) shRNA-transfected 8226 cells treated +/−thapsigargin (Tg in uM) or dox to induce DEPTOR knockdown, followed by immunoblot assay; D) DEPTOR shRNA-transfected 8226 cells treated +/− dox and +/− increasing concentrations (uM) of JNK inhibitor, followed by immunoblot; E)Same cells as in ‘D’ treated +/− bortezomib +/− JNK inhibitor; F) DEPTOR shRNA-transfected cells treated with dox or bortezomib and increasing concentrations of SP600125 JNK inhibitor, followed by apoptosis assay. Data are means+/− SD, n=3; *=different from control (no inhibitor), p<0.05.

Another potential pathway of MM cell injury induced by DEPTOR knockdown is ER stress due to the hypothesized acute increase in protein translation that would occur from TORC1 stimulation. Assays for lambda light chain protein expression in 8226 cells induced to silence DEPTOR did not support this hypothesis as, at least in short term cultures in which apoptosis is induced up to 72 hrs, there was no significant increase in synthesis (suppl fig 2). To further investigate this issue, we tested several pathways of the unfolded protein response which are activated subsequent to ER stress in MM cells [9]. As shown in fig 4A, conditions of DEPTOR KD which induced apoptotic PARP cleavage and caspase 3 cleavage, had no effect on phosphorylation of IRE-1, phosphorylation of eIF-2α, or induction of CHOP. As a positive control, fig 4B demonstrates that bortezomib, which induces ER stress via its proteasome inhibition, can activate these ER stress/UPR markers in these 8226 cells. An additional marker for ER stress-induced apoptosis recently identified is stimulated expression of the pro-apoptotic DR5 death receptor [10]. As shown in fig 4C, DEPTOR knockdown did not affect DR5 levels while the ER stress inducer thapsigargin (Tg at 0.5 or 1 uM), used as a positive control, successfully enhanced expression. DR5 was also induced by bortezomib in the same cells (not shown).

Phosphorylation of JNK also participates in one of the UPR pathways, becoming activated downstream of IRE-1 [11]. Although IRE-1 was not activated by DEPTOR KD, JNK became specifically phosphorylated by DEPTOR silencing (Fig 4A). JNK activation also may participate in bortezomib-induced apoptosis of MM cells [12]. We, thus, tested if JNK activation was relevant to DEPTOR KD-induced apoptosis by exposing MM cells to the SP600125 JNK inhibitor during doxycycline-induced KD. As shown in fig 4D and 4E, inhibition of JNK activity was demonstrated in DEPTOR-KD cells exposed to dox or the same cells exposed to bortezomib by a decrease in JNK-mediated phosphorylation of c-jun. Inhibition was almost complete when the concentration of SP600125 reached 20 uM. As shown in fig 4F, the JNK inhibitor had no effect on MM cell apoptosis induced by DEPTOR KD although it significantly decreased bortezomib-induced apoptosis as had been previously described [12]. Collectively, these results rule out the possibility that ER stress and UPR activation mediate apoptosis during DEPTOR KD in MM cells. In fact, the absence of eIF-2α phosphorylation, IRE-1 phosphorylation and CHOP induction suggest that induction of JNK phosphorylation during DEPTOR silencing is not due to ER stress but mediated by an independent mechanism, possibly secondary to AKT down-regulation as it has been previously reported [13] that AKT negatively regulates the ASK-1/JNK cascade.

### P53-independent upregulation of p21 and p27 expression occurs secondary to DEPTOR KD

**Figure 5:**
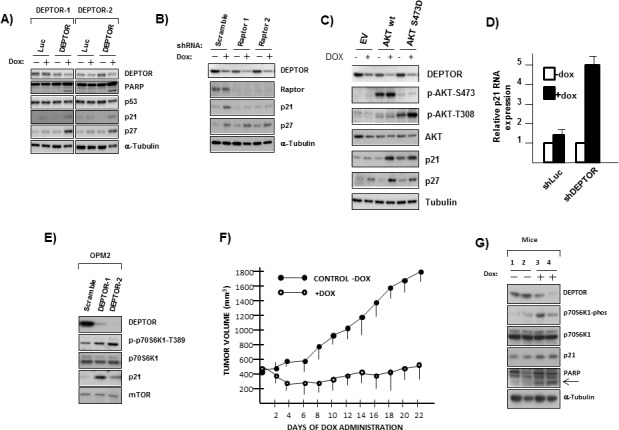
DEPTOR knockdown-induces P21 and P27 in a P53-independent fashion A) Transfected cells treated +/− dox followed by immunoblot. B) Inducible shDEPTOR cells ectopically expressing scramble or RAPTOR shRNAS treated +/− dox followed by immunoblot; C) Inducible shDEPTOR cells ectopically expressing EV or AKT(WT) or phosphomimetic AKT treated+/− dox followed by immunoblot; D) qt-PCR for p21 RNA expression following infection with control (shLuc) or DEPTOR shRNA and treated+/− dox (mean+/− SD, n=3). E) Acute infection with shRNA scramble (control) or DEPTOR shRNA in OPM-2 cells followed by immunoblot; F) Mice (8/group) challenged with SQ injection of inducible 8226 cells and, when tumor size reached 400 mm^3^, mice were randomized to receive dox in water or no dox. Tumor size is mean+/−SDs. G) Western blot of tumor protein extracted from 2 separate mice from each group.

A previous publication [14] demonstrated that activation of mTORC1 could induce tumor cell growth arrest via upregulation of p53 protein translation. Since DEPTOR KD in MM cells induced apoptosis via a TORC1-dependent pathway, we tested effects on p53 expression. Although 8226 cells contain mutated p53 (15), a previous study [16] indicated that p53 could activate BAX and induce apoptosis in the absence of transcriptional activity. Figure 5A, however, demonstrates that the two DEPTOR KD shRNAs had no significant effect on p53 expression though clearly inducing p21/p27 expression. When DEPTOR KD occurs in RAPTOR-silenced cells, the p21 upregulation is prevented although p27 upregulation is maintained (fig 5B). Since RAPTOR KD protects against apoptosis/cell cycle arrest (fig 3), these data argue against a role for p27 in the anti-MM response. However, p21 could be relevant. In addition, when DEPTOR is silenced in AKT over-expressing MM cells (fig 5C), the p21 upregulation is maintained also supporting the hypothesis that it is relevant to the apoptotic response. Quantitative real time PCR (fig 5D) demonstrates that p21 is upregulated at the RNA level by DEPTOR knock down. P21 upregulation following DEPTOR knockdown also occurred in OPM-2 MM cells (Fig 5E) which were adversely affected (as shown in fig 1). P21 upregulation also occurs during tumor regression *in vivo* when DEPTOR is silenced. In these latter experiments, 8226 cells containing the inducible DEPTOR shRNA were injected subcutaneously in NOD/SCID mice. When tumors reached 400 mm3 in size, mice were randomized to receive doxycycline in their water or to a control group. Ingestion of doxycycline had a very clear cytoreductive effect with complete prevention of tumor growth (Fig 5F). In the absence of dox, tumors grew normally. At day+5 after initiation of doxycycline ingestion, 2 mice from each group were sacrificed and their tumors were harvested for western blot. As shown in figure 5G, DEPTOR knockdown was associated with upregulation of p70 phosphorylation, p21 expression and PARP cleavage. Thus, the pathway of DEPTOR knockdown leading to TORC1 activation, enhanced p21 expression and tumor regression is recapitulated *in vivo*.

### p21 silencing prevents the deleterious effects of DEPTOR knockdown

**Figure 6:**
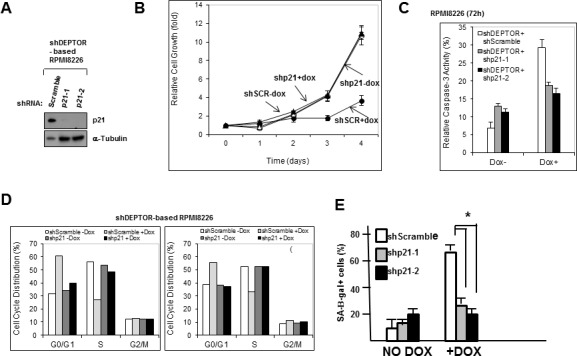
P21 upregulation induced by DEPTOR knockdown mediates Apoptosis and cell cycle arrest A)P21 expression in inducible 8226 cells transduced with scramble shRNAs or shRNAs targeting p21; B)MTT assay showing growth (over time) in same cells as in ‘A’ treated +/− dox. Data are means+/−SD, n=3; C) Apoptosis assay in transfected cells treated+/− dox. Data are means+/−SD, n=3. P21 silencing significantly (p<0.05) inhibited dox-induced apoptosis; D) Cell cycle analysis in same cells. Data are means of 3 separate experiments. E) Senescence assay in same cells. Data are means+/−SD, n=3; *=different by p<0.05.

To test the role of p21 in DEPTOR-KD-induced MM cell cytoreduction, we silenced p21 as shown in fig 6A. An MTT assay comparing one of the p21-knocked down lines to a control transfected with lentivirus targeting a scrambled sequence (SCR) demonstrated that p21 knockdown prevented the cytoreduction induced by DEPTOR silencing from dox treatment (fig 6B). Further support for the importance of p21 upregulation is shown in apoptosis assays (fig 6C) where doxycycline induced significant apoptosis in SCR-transfected control cells which was greatly reduced in these cells when expressing p21 shRNA. The same is true for cell cycle analysis (fig 6D). As shown, G1/S arrest is only induced upon treatment with dox in SCR-transfected control cells while p21 silencing (shp21-1, left panel;shp21-2, right panel) prevents cell cycle arrest. The induction of senescence by DEPTOR KD is also prevented by p21 silencing (fig 6E). Collectively, these data implicate the induction of p21 expression in all the identified adverse effects on MM cells that follow DEPTOR silencing.

### P21 upregulation may be mediated by effects on miRNAs

**Figure 7:**
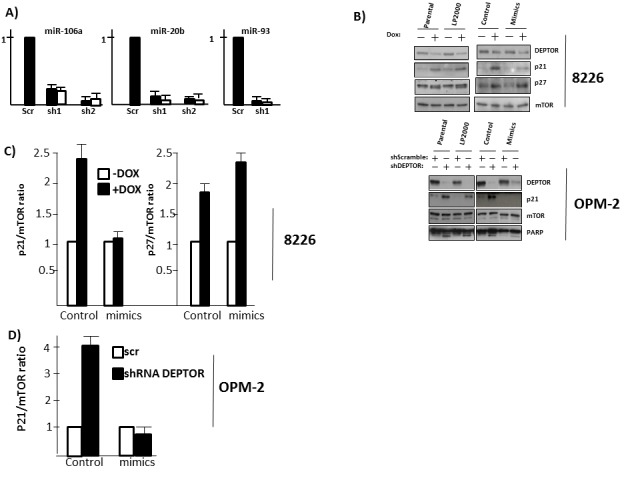
Role of miRNAs A) qt-PCR assessment in 2 MM cell lines of expression of 3 miRNAs of the 106b family following DEPTOR silencing by acute infection with 2 independent shRNAs (black bars=8226; white bars=OPM-2); B) Dox-inducible 8226 cells (upper panel) not treated (parental), treated with Lipofectamine 2000 (LP2000) alone, transfected with control mimics (control) or mimics of all 3 miRNAs (mimics). Cells then exposed to dox to silence DEPTOR followed by immunoblot. OPM-2 cells (lower panel) similarly treated followed by acute infection with control or shRNA targeting DEPTOR; C) The experiments in ‘B’ were repeated 3x and p21/mTOR or p27/mTOR ratios determined by densitometry. Data show mean+/−SD.

**Figure 8:**
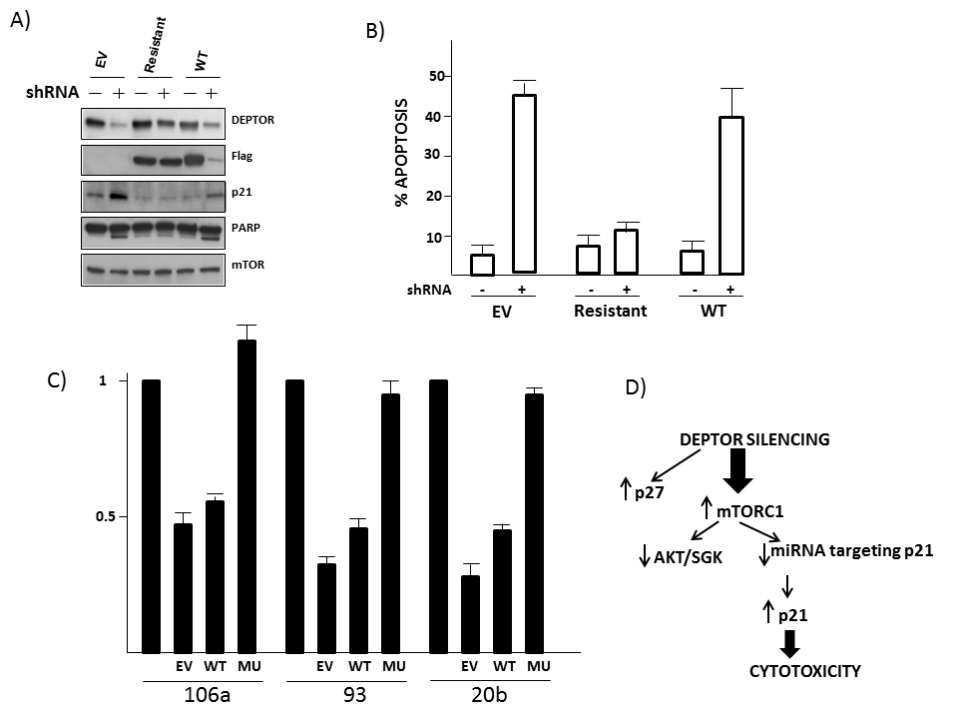
Use of shRNA-resistant DEPTOR A) 8226 MM cells transfected with empty vector (EV), mutant DEPTOR (resistant) or wild type (WT) DEPTOR and either treated with or without shRNA targeting DEPTOR, followed by immunoblot; B) % apoptosis (mean+/− SD, n=3) following DEPTOR knockdown (+/−shRNA) in EV, mutant-DEPTOR (resistant) or wild type DEPTOR-transfected 8226 cells. C) qt-PCR quantification of relative amounts of miRNA 106a, 93 and 20b in MM cells transfected as in ‘A’ (EV=empty vector; WT=wild type DEPTOR; Mu=mutant (resistant) DEPTOR). Data are means+/−SD, n=3. D) Model of events following DEPTOR knockdown in MM cells.

The above results in fig 5 indicated that p21 upregulation following DEPTOR KD could not be explained by effects on p53 or simply by TORC1-mediated stimulation of p21 translation (ie., qt-PCR assay for p21 RNA). Since mTOR activity can affect miRNA levels [17, 18] and since miRNAs of the 106b family can regulate p21 expression [19], we investigated effects on members of this miRNA family. Initial experiments utilizing U6 as an endogenous control indicated that addition of doxycycline to induce DEPTOR shRNA non-specifically decreased qRT-PCR determinations. Thus, we knocked down DEPTOR in 8226 or OPM-2 cells by acute infection with control (scrambled (Scr) sequence) or DEPTOR shRNA targeting the two DEPTOR sequences used in figure 1A. miRNA expression was then normalized to RNU 49. As shown in fig 7A, DEPTOR KD achieved by both shRNAs resulted in downregulation of miRNA-106a and miR-20b in both cell lines. In contrast, downregulation of miRNA-93 was achieved by shRNA1 but not shRNA 2. Since shRNA2 was, in fact, more effective than shRNA1 in silencing DEPTOR (see fig 1), this suggested the effects on miRNA-93 could have been due to off-target effects. However, use of a shRNA-resistant construct argued against off-target effects on miRNA-93 (see below and fig 8). Additional qRT-PCR determinations demonstrated that down-regulation of miRNA 106b was also achieved by shRNA1 but not shRNA2 (not shown). However, experiments with the shRNA-resistant DEPTOR construct (below) could not rule out that this response in miRNA106b was possibly due to off-target effects. It is unclear why shRNA2 was incapable of inhibiting expression of miRNA-93 and 106b. One possibility is that it relates to the specific locus on chromosome 7 in which these two miRNAs reside. For example, effects of shRNA2 may include non-specific effects on a transcription factor specific to the miR-106b/93 cluster on this chromosome. This question is under further investigation.

To next test whether the downregulation of miRNA 106a, 20b and 93 was instrumental in stimulation of p21 expression, we transfected a pool of mimics representing these 3 miRNAs into the dox-inducible 8226 line and tested p21 protein expression following DEPTOR KD. In preliminary experiments testing various transfection reagents, Lipofectamine 2000 was the most effective in terms of transfection efficiency for mimic expression. Thus, we used lipofectamine 2000 which consistently gave a transfection efficiency of 60-70%. In fig 7B (upper panel) we added dox to parental 8226 cells, to 8226 cells treated only with lipofectamine 2000 (LP 2000), to cells transfected with equal amounts of control mimics (control) and to cells transfected with mimics of the above miRNAs (mimics). As shown, p21 upregulation was specifically inhibited by the miRNA mimics. In contrast, there were no effects on the dox-induced p27 upregulation attesting to the specificity of the mimics. We similarly tested OPM-2 cells ectopically expressing the mimics with DEPTOR knockdown induced by acute infection with lentivirus. In similar fashion, mimic expression in OPM-2 cells also prevented the p21 upregulation. Fig 7C and 7D shows means+/−SD of 3 separate experiments for both cell lines. The pool of miRNA mimics used in these experiments contained each individual mimic at 100nM. We also performed follow-up experiments testing each mimic separately, used in concentrations from 100-400nM, but could not reproduce the effects on p21 expression seen when the pool was used (not shown). This is not too surprising as miRNAs are known to act cooperatively. Based on the typical seed-mediated regulation, the three miRNAs would compete for the same binding site on the target as they share the seed sequence. However, it is possible that non-canonical binding beyond the seed has allowed additional effects of the individual miRNAs. In fact, sequence analysis of the p21 3′UTR shows that each of the miRNAs has 1-2 additional regions of 7-9nt identity beyond the seed region.

The mimics also prevented the apoptosis induced by DEPTOR knockdown. The percent apoptosis in 8226 cells due to DEPTOR knockdown was reduced from 54+/−5% to 24+/−3% and, in OPM-2 cells, from 47+/−6% to 21+/− 5% (mean+/−SD of 3 experiments). The immunoblot assay for PARP cleavage in OPM-2 cells (bottom panel, fig 7B) also supports the ability of the mimics to diminish apoptosis.

To further support the involvement of miRNAs in this pathway and rule out any off-target effects of DEPTOR shRNA on the miRNAs (especially since only 1 of the shRNAs downregulated miRNA-93), we engineered a shRNA-resistant DEPTOR construct. MM cells were transfected to stably express empty vector (EV), tagged wild type DEPTOR (WT) or mutant resistant DEPTOR and then DEPTOR was silenced with shRNA. As shown in the fig 8A immunoblot, the mutant construct was resistant to DEPTOR shRNA and rescued cells from induction of p21 and PARP cleavage. Similarly, apoptosis assessed by flow analysis demonstrated rescue by the shRNA-resistant DEPTOR (fig 8B). Finally, the shRNA-resistant DEPTOR also rescued 8226 cells from the inhibited expression of miRNA 106a, 93 and 20b (fig 8C). These data further support the notion that DEPTOR knockdown specifically inhibits miRNA expression and upregulates p21 expression associated with apoptosis, thus ruling out off-target effects. A hypothesized model is presented in fig 8D.

## DISCUSSION

The results of this study indicate that DEPTOR silencing induces adverse effects on MM cells via a novel TORC1-miRNA-p21 cascade. Several of these miRNAs had previously been identified as being regulated by mTORC1 [17]. Their MTORC1-dependent down regulation is key to the critical effects on p21 expression. In contrast, we found no evidence that a feedback inhibition of the PI3K-AKT survival pathway or an induction of acute ER stress was relevant to the cytotoxic response of MM cells following DEPTOR KD.

There are many previous studies [20-22] attesting to the regulatory role of p21 in MM cell viability and growth supporting its importance. Our data support the hypothesis that acute activation of mTORC1 induces MM cell death via upregulation of this CDK inhibitor. As mentioned by Peterson [1], it is notable that development of plasmacytomas in mice is facilitated by genetic loss of mTOR function [23], suggesting mTORC1 can be a tumor suppressor in the MM model.

The importance of p21 in the cytotoxic pathway may also explain the paradox of how MM cell TORC1 activation can be lethal in one condition and promote growth expansion in another. For example, following stimulation with the MM growth factor IL-6, MM growth expansion occurs which is, at least partially, mediated by activation of mTORC1 [24]. However, IL-6 also significantly inhibits p21 expression [20]. Thus, this differential effect on p21 expression may explain the inconsistency of effects when TORC1 is stimulated via DEPTOR knockdown versus cytokine stimulation. In the former, the ability of TORC1 activation to stimulate p21 expression results in lethality; In the latter, TORC1 activation (induced by IL-6) promotes mTOR-dependent growth expansion because IL-6 does not allow p21 upregulation. Whether IL-6's effect on p21 expression is mediated via effects on the miRNA 106b family is an interesting question and will be studied in future experiments.

It has also been hypothesized that DEPTOR is especially critical in MM cells because the huge demand for Ig synthesis sensitizes them to ER stress [1]. In this hypothesis, DEPTOR over-expression is protective by restraining TORC1 and cap dependent translation, thus containing ER stress. The MM models we used synthesize significant levels of Ig and are known to be under basal levels of ER stress [9]. In addition, a recent report by Kato et al [25] describes an apoptotic pathway induced by ER stress and mediated by a subsequent activation of TORC1 which reduced AKT phosphorylation/activation directly leading to activation of IRE-1/JNK and apoptosis. However, although MM apoptosis in our study was also mediated by TORC1 and associated with AKT downregulation, prevention of AKT downregulation did not affect 8226 lethality, there was no increase in ER stress markers which included IRE-1 activation and prevention of JNK activation also had no effect. These data suggest ER stress did not play a role in the cytotoxicity observed. One caveat to this interpretation is that our assays were all performed after short term culture following DEPTOR knockdown. It is certainly possible that targeting DEPTOR for longer intervals could result in a significant increase in cap-dependent translation, Ig synthesis, ER stress and death. The additional potential adverse effects of AKT/SGK inhibition and p27 upregulation may also contribute to MM cell fate over longer intervals. Nevertheless, the model explaining anti-MM effects in this study, as shown in fig 8D, delineates a novel DEPTOR-TORC1-miRNA106b-p21 pathway determining fate of MM cells. As suggested by Peterson et al [1], it is conceivable that small molecule inhibitors could be developed to target DEPTOR in MM, by interrupting the binding of the DEPTOR PDZ domain to mTOR. If successful in development, such a therapeutic could be monitored by early effects on tumor cell p21 expression which could predict subsequent clinical outcome.

## MATERIALS & METHODS

### Cell lines, plasmids, transfections

The MM and HCC cell lines were obtained from ATCC. The short hairpin RNAs (shRNA)/pLKO.1 targeting raptor and the control scrambled sequence were obtained from Addgene as previously described [26]. HA-Akt gene was amplified by PCR from plasmid pDONR223-AKT1 (Addgene plasmid 23752) with inclusion of the HA sequence in the 5′ primer to generate HA-Akt, and HA-Akt was subsequently subcloned into pLenti6. The phosphomimetic AKT S473D was created from pLenti6 HA-Akt plasmid with QuickChange XL Site-Directed Mutagenesis Kit (Agilent Technologies) according to manufacturer's protocol. The inducible DEPTOR shRNA used the following sequences: Human DEPTOR miRshRNA target sequence 1: 5′-GCCATGACAATCGGAAATCTA-3′; Human DEPTOR miRshRNA target sequence 2: 5′;-GCAAGGAAGACATTCACGATT-3′;; Luc miRshRNA control target sequence 5′;-CCGCCTGAAGTCTCTGATTAA-3′;; GFP miRshRNA control target sequence 5′;-CAAGCTGACCCTGAAGTTCAT-3′;; shRNAs were subcloned respectively into the pEN_TmiRc3 (#25748, Addgene) entry vector, and then transferred into the pSLIK-Neo lentiviral vector (#25735, Addgene) by gateway recombination. An shRNA-resistant DEPTOR construct was generated via creation of four nucleotide synonymous mutations (from ATCT to GAGC, 771-774) within the shRNA target region. These mutations were chosen so as to not alter the amino acid sequence of the mutated DEPTOR. Lentivirus was produced by the UCLA Vector Core facility and stable cell lines were made by transducing cells with lentivirus and selecting with geneticin. Lentiviral infection of MM cells was performed as previously described [27]. Knockdown was induced by addition of doxycycline (Dox) in the culture media at a final concentration of 1ug /ml.

### Cytotoxicity assays

Apoptosis was assayed by staining for activated caspase-3 (BD BioSciences) and assessed using flow cytometry as previously described [28]. MTT survival and cell cycle analysis were assessed as previously described [27, 28]. The percent of cells induced into senescence was enumerated as previously described [29] by assaying cell-associated beta galactosidase activity at pH 6.

### Xenograft experiments

8226 cells (10^7^/mouse) were injected SQ admixed with matrigel into NOD/SCID mice as described [30]. When tumor volume reached 400mm3, mice were randomized to receive drinking water containing doxycycline (+dextrose) or no dox (dextrose alone). Tumor volume was calculated as previously described [30]. In addition, 2 tumors from dox-treated mice or non-treated mice were randomly chosen and excised for Western blot analysis on protein extracts as previously described [30].

### Evaluation of protein and RNA expression

Western blot was performed as described [7,29,30]. Real time PCR for p21 RNA and GAPDH RNA was performed as described [7,27,28]. Briefly, gene amplifications for real time PCR were performed with an ABI PRISM 7700 sequence detection system. Each 20 μL reaction in a 96-well plate comprised 9 μL of cDNA template, 1 μL of 20x primer mixtures for p21 or GAPDH and 10 μL 2xTaqman Universal PCR Master mixed with AmpErase^®^ UNG. After an initial 2 minute at 50 °C to activate ampEras^e®^ and a denaturation step of 10 min at 95°C, 60 cycles of amplification were performed with denaturation for 15 s at 95°C and annealing/extension for 1 minute at 60°C. All samples were run in triplicate and no template controls were included in all plates for both p21 and GAPDH. AKT *in vitro* kinase assays were performed as previously described [7].

### miRNA

miRNA expression was quantified by qRT-PCR as previously described [17]. Expression was normalized to small nuclear RNA (snRNA) RNU49. Assays were performed using three biologic replicates and three technical replicates for each treatment condition. Only one PCR product was observed for each assay. RNA duplexes corresponding to mature miRNAs of the miR-106b family and negative control mimics were purchased from Thermo Scientific and used as previously described [31]. They were transfected using Lipofectamine 2000 as previously described [19]. Transfection efficiency was 60-70%. Ig ELISA-Ig ELISA assays were performed on lysates from 8226 cells using the Human Lambda ELISA Quantification SET (Bethyl Laboratories, cat #E80-116).

### Statistics

Quantitative increases or decreases in protein phosphorylation on Western blots were evaluated by densitometric analysis. All Western blots were repeated at least 3 times. The t-test was used to determine significance of differences between groups.

## SUPPLEMENTARY MATERIAL FIGURES


