# Supplementary material for: DEPTOR is linked to a TORC1-p21 survival proliferation pathway in multiple myeloma cells
Source: Genes Cancer. 2014 Nov;5(11-12):407–19. doi: 10.18632/genesandcancer.44 (PMC4279438; doi:10.18632/genesandcancer.44)
Supplement: Supplementary file 1 [file ganc-05-407-s001.pdf]

DEPTOR is linked to a TORC1-p21 survival proliferation pathway in multiple myeloma cells

Supplementary Material

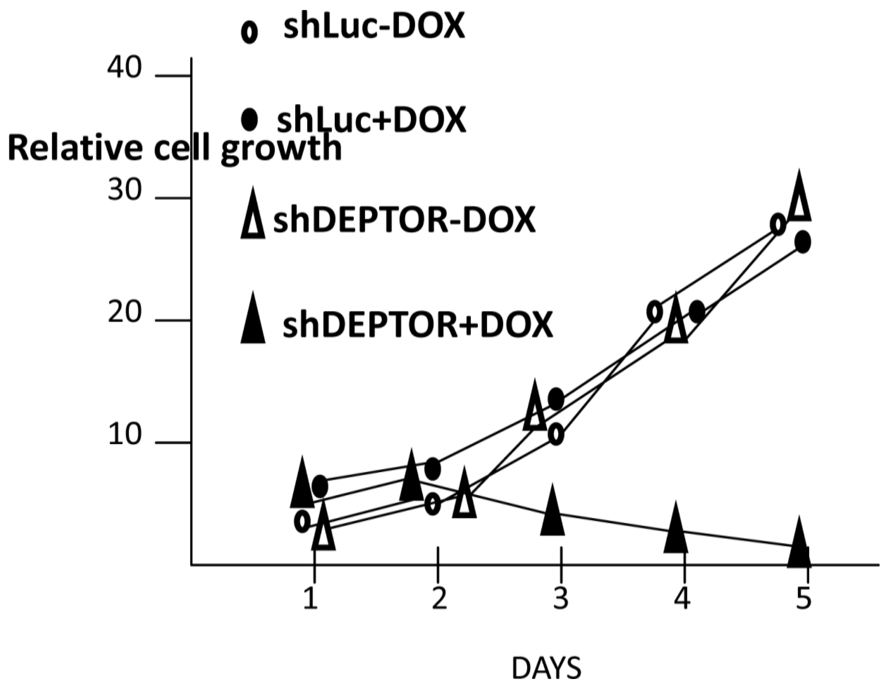

Suppl fig 1: MTT assay in 8226 cells expressing either inducible shRNA DEPTOR against target 2 or control (shLuc). Cells are either treated +/- dox to induce shRNA expression. MTT assay performed after 1, 2, 3, 4 or 5 days of dox exposure(+DOX) or no dox (-DOX).

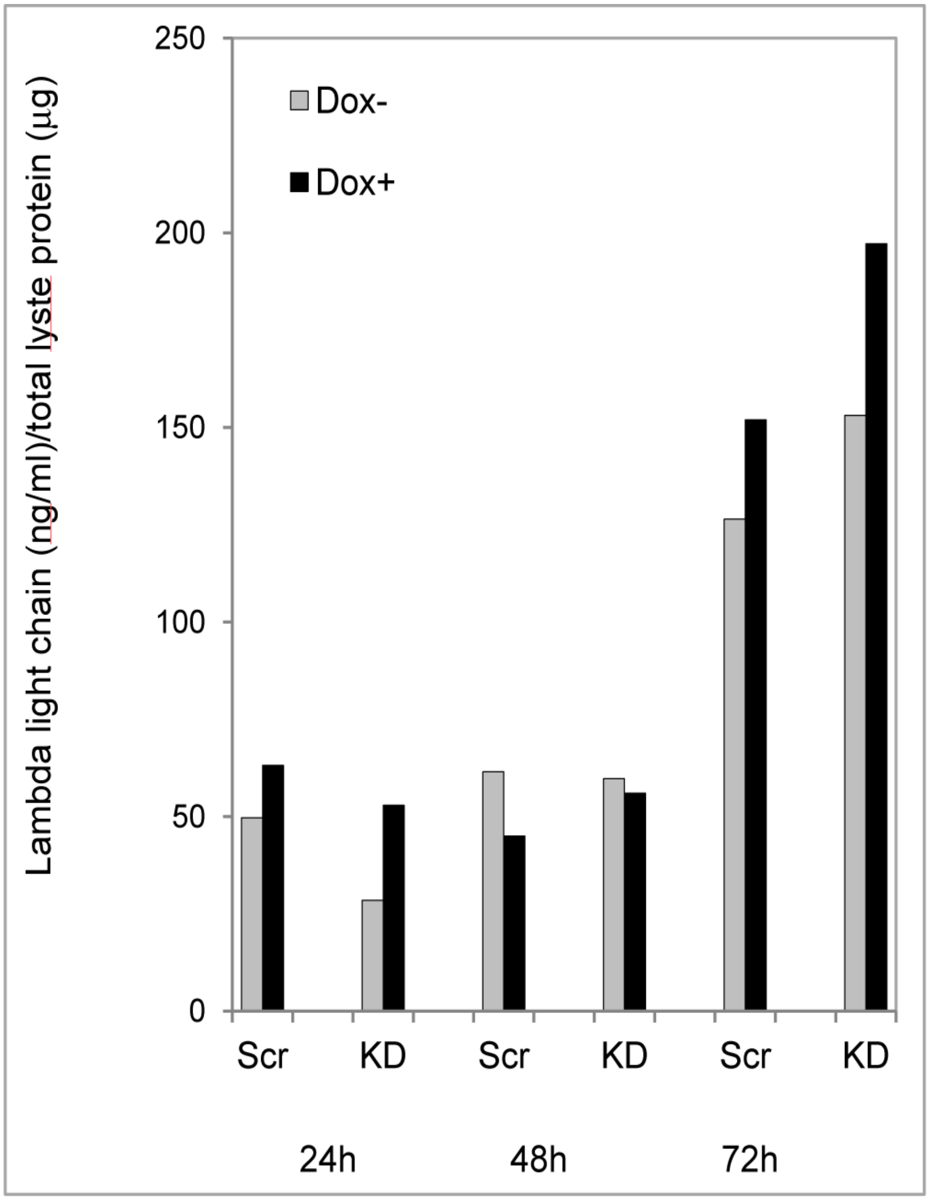

Suppl fig 2: Ig lambda expression in 8226 cell lysates (ng/ml/ug protein). Cells were transfected with either inducible shRNA to DEPTOR (Knockdown (KD))or control (Scr). Cells were treated +/- dox to induce shRNA expression for 24, 48 or 72 hrs. Data are means of 2 experiments (each experiment had 4 replicates). There are no significant differences between the groups.
